# A case of successful revascularization and renal recovery after 6 days of renal artery occlusion

**DOI:** 10.1016/j.jvscit.2024.101453

**Published:** 2024-02-12

**Authors:** Muner M.B. Mohamed, Khalid M.G. Mohammed, MoetazBellah S.A. Kadoura, W.Charles Sternbergh, Juan Carlos Q. Velez

**Affiliations:** a Department of Nephrology, Ochsner Health System, New Orleans, LA; b Ochsner Clinical School, The University of Queensland, Brisbane, QLD, Australia; c Faculty of Medicine, Tanta University, Tanta, Egypt; d Faculty of Medicine, Tobruk University, Tobruk, Libya; e Division of Vascular and Endovascular Surgery, Ochsner Health, New Orleans, LA

**Keywords:** Renal artery occlusion, Thrombolysis

## Abstract

A 72-year-old man with peripheral arterial disease, an atrophic left kidney, and prior right renal chimney stent as part of a complex endovascular abdominal aortic aneurysm repair presented to our emergency department with right flank pain and anuria resulting from right artery occlusion. His serum creatinine on admission was 7.5 mg/dL. Computed tomography angiography 6 days after the onset of his symptoms revealed complete occlusion of the right renal artery stent. Percutaneous thrombectomy was performed restored renal blood flow. The urine flow started the following day, and his serum creatinine decreased to 3.5 mg/dL 7 days after discharge.

Acute renal artery occlusion (RAO) can lead to irreversible loss of function of the affected kidney. Its diagnosis can be delayed or overlooked when evaluating impaired renal function due to it is nonspecific presentation.^1^ A general consensus has recommended that revascularization should be attempted as early as possible for successful renal function recovery.^2^ Although early diagnosis and treatment of acute RAO reduce ischemic kidney injury, the benefit of intervention after prolonged ischemia is controversial.^3^ The patient provided written informed consent for the report of his case details and imaging studies.

## Case report

**Fig 1:**
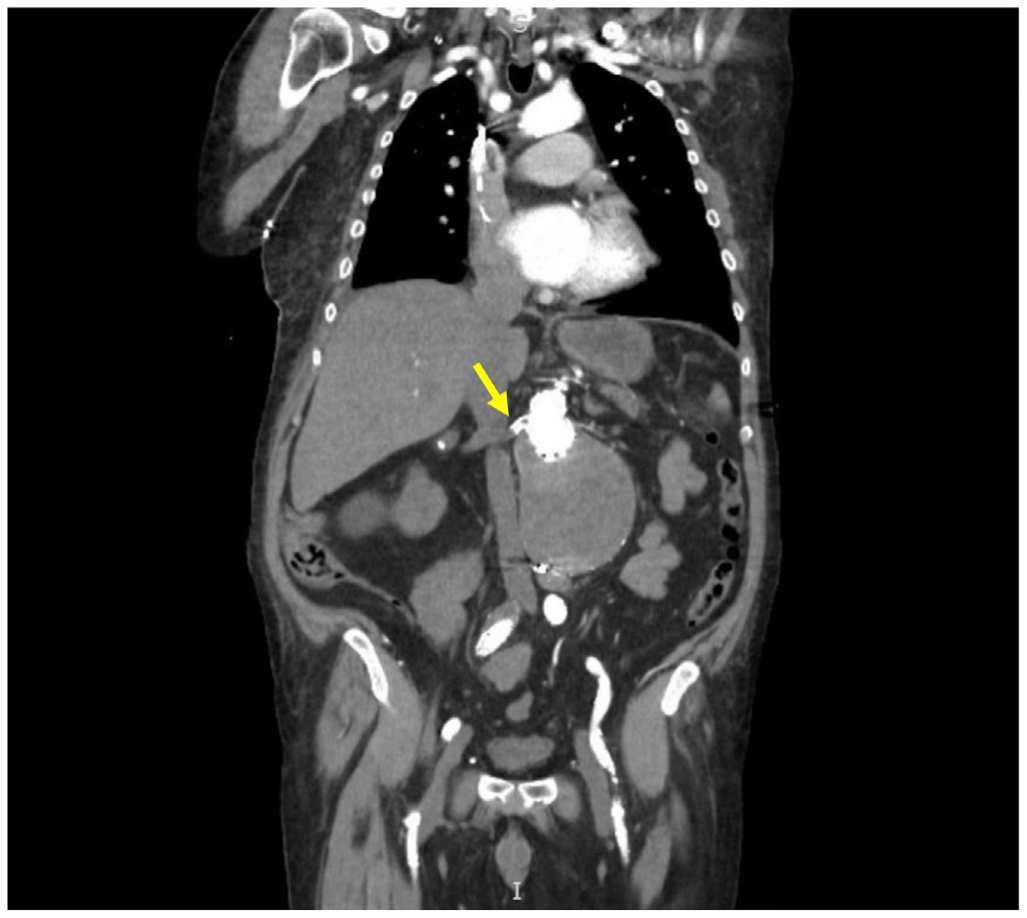
A computed tomography scan with intravenous contrast medium of the abdomen showing an occluded proximal right renal artery (*yellow arrow*).

**Fig 2:**
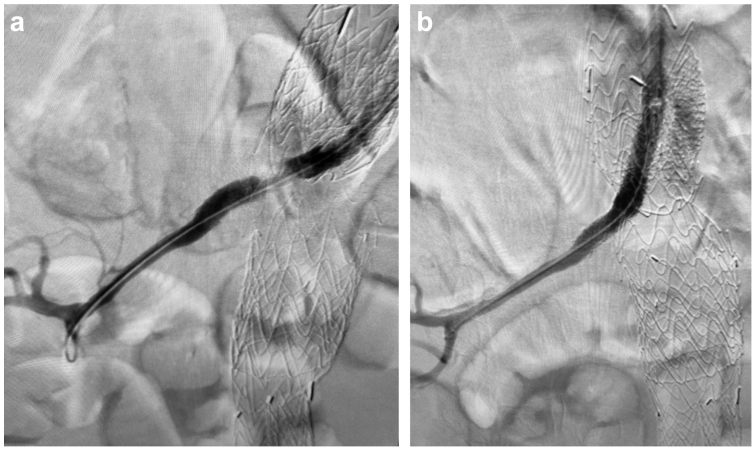
After percutaneous mechanical thrombectomy, before (**A**) and after (**B**) stent placement.

A 72-year-old man was admitted to our hospital for right flank pain and anuria. He had a medical history of peripheral arterial disease, chronic kidney disease stage 3, a right kidney cyst, hypertension, diabetes mellitus type 2, atrial fibrillation, coronary artery disease, and hyperlipidemia. He had undergone endovascular aneurysm repair 12 years before his current presentation. He presented with a symptomatic type Ia endoleak 3 years before the current presentation and underwent urgent complex revision with parallel stents (“chimneys”) to the right renal and superior mesenteric arteries and proximal extension of the aortic endograft. Due to its diminutive size, the left renal artery was intentionally sacrificed. On presentation, his medications included atorvastatin, amlodipine, apixaban, clopidogrel bisulfate, losartan, and metoprolol succinate. In the emergency department, the patient stated the pain started 4 days before arrival. He also noted a decrease in urine output during the same period, with complete absence of urination 2 days before presentation. He denied any other symptoms. The physical examination revealed a temperature of 98.1°F (36.7°C), blood pressure of 197/98 mm Hg, heart rate of 92 bpm, and respiratory rate of 18 breaths/min. The abdomen was very tender to palpation over the right flank, and the patient had bilateral lower extremity pitting edema. The remainder of the examination was normal. He was noted to have an elevated serum creatinine (sCr) of 7.5 mg/dL. His last sCr before admission was 2.0 mg/dL (1 month prior). The laboratory data are presented in the Table. A nicardipine infusion was started due to concerns of a hypertensive emergency. A computed tomography scan without contrast for renal stones in the emergency department showed postoperative changes after endovascular aneurysm repair with enlargement of the excluded aneurysmal sac from 7.9 cm to 9.3 cm, suggesting an endoleak. Vascular surgery recommended renal Doppler ultrasound. The Doppler ultrasound scan done on the day of admission revealed a large, right, simple cyst approximately 8.6 cm in size, patent right renal vasculature, and an atrophic left kidney. At this time, no vascular intervention was planned, and nephrology was consulted to evaluate the etiology of his acute kidney injury. A temporary dialysis catheter was placed on the second day of admission in anticipation of the need for dialysis. One of us and an independent experienced sonographer reviewed the source imaging from the renal artery duplex ultrasound examination. Due to poor imaging windows and challenges in having the patient hold his breath, the study should have been considered nondiagnostic. Based on the patient's 3-day history of right flank pain and 2 days of anuria at the time of presentation, computed tomography angiography (CTA) was performed on the third day of admission. It revealed no endoleak but showed a proximal right renal artery stent with complete occlusion of the proximal right renal artery (Fig 1). The patient received hemodialysis after the CTA. Although the likelihood of meaningful recovery of renal function after 6 days of ischemia was thought to be quite low, the plan was discussed with the patient who agreed to undergo percutaneous thrombectomy and stent placement, which were performed on the third day of admission. The operative findings revealed a thrombosed right renal stent (7 × 59-mm VBX stent; W.L. Gore & Associates) secondary to external compression. Thrombus was present, which was cleared with AngioJet thrombectomy (Boston Scientific). The stenosis resolved with placement of a 7 × 39-mm Omnilink balloon-expandable stent (Abbott Cardiovascular; Fig 2). During the following 12 hours, he voided 150 mL of urine. A second session of hemodialysis was performed for metabolic clearance. On the fourth day of admission, the daily urine output was 2 L. The patient did not require additional dialysis and was discharged home. At his follow-up in our nephrology clinic 1 week after discharge, his sCr was 3.5 mg/dL. One month later, his sCr was 3.2 mg/dL.

**Table:** Laboratory data at admission, discharge, and follow-up

Parameter	Normal value	On admission (10/18/2023)	On discharge (10/26/2023)	On follow-up	
				11/02/2023	12/04/2023
Clinical chemistry					
Sodium	136-145 mmol/L	136	140	136	138
Potassium	3.5-5.1 mmol/L	4.7	3.2	3.4	4.6
Chloride	95-110 mmol/L	108	101	94	107
Bicarbonate	23-29 mmol/L	12	23	26	23
Anion gap	5-15 mmol/L	16	16	16	8
Blood urea nitrogen	6-20 mg/dL	58	53	48	36
Creatinine	0.5-1.4 mg/dL	7.5	8.1	3.5	3.2
eGFR	>60 mL/min/1.73 m^2^	7.1	6.5	18	19.7
Calcium	8.7-10.5 mg/dL	8.5	8.1	9.6	9.1
Glucose	70-110 mg/dL	117	100	122	100
Phosphorus	2.7-4.5 mg/dL	6.4	6.5	3.6	5
Magnesium	1.6-2.6 mg/dL	2	1.9	–	–
Alkaline phosphatase	55-135 U/L	99	114	–	–
Protein total	6.0-8.4 g/dL	6.8	6.3	–	–
Albumin	3.5-5.2 g/dL	3.1	3.1	3.1	3.1
Bilirubin total	0.1-1.0 mg/dL	0.9	0.7	–	–
AST	10-40 U/L	14	35	–	–
ALT	10-44 U/L	11	31	–	–
Complete blood count					
Hemoglobin	14.0-18.0 g/dL	9.3	8.0	–	–
Platelet count	150-350 K/μL	209	201	–	–
WBC count	3.90-12.70 K/μL	22	8	–	–
Urine					
Color	Yellow, straw, amber	Yellow	–	–	–
Appearance	Clear	Cloudy	–	–	–
Specific gravity	1.005-1.030	1.010	–	–	–
pH	5.0-8.0	8.0	–	–	–
Glucose	Negative	Negative	–	–	–
Protein	Negative	3+	–	–	–
Ketones	Negative	Negative	–	–	–
Occult blood	Negative	2+	–	–	–
Nitrite	Negative	Negative	–	–	–
Bilirubin	Negative	Negative	–	–	–
Leukocytes	Negative	1+	–	–	–
RBCs	0-4 RBCs/HPF	26	–	–	–
WBCs	0-5 WBCs/HPF	21	–	–	–
Bacteria	None or occult/HPF	Negative	–	–	–

*ALT,* Alanine transaminase; *AST,* aspartate transaminase; *eGFR,* estimated glomerular filtration rate; *HPF,* high-power field; *RBC,* red blood cell; *WBC,* white blood cell.

## Discussion

RAO is an uncommon vascular complication that carries a significant risk of renal failure. The risk factors for RAO include atherosclerosis, renal artery dissection, iatrogenic thrombosis after vascular intervention, congenital horseshoe kidney, embolus to the kidney, and procoagulant states.^4^ The presentation is nonspecific; thus, making an early diagnosis very challenging. In a study by Korzets et al,^5^ the time to diagnose RAO was from 24 hours to 6 days, and 9 of the 11 patients with RAO were considered to have a diagnosis other than RAO. Most patients present with flank pain. Others can present with hematuria, hypertensive crisis, acute renal failure, or anuria when the occlusion is bilateral or in a solitary kidney.^3^^,^^4^ Doppler ultrasound can be used as an initial tool to diagnose renal RAO when the kidney is >8.5 cm long. However, the results are operator dependent.^6^ In our case, the Doppler ultrasound scan was thought to be an error because it read as the right renal artery to be widely patent with normal flow. CTA provides superior sensitivity to identify RAO. Supporting evidence for specific treatment of acute RAO is lacking and is based mainly on small series and case reports. The available treatment options include conservative treatment with anticoagulation, surgical thrombectomy, and percutaneous thrombolytic techniques.^3^^,^^4^ The benefits of intervention include recovery of renal function, patency of the occluded vessel, and relief of hypertension.^7^ Despite the improvement in these techniques, the risk includes complications such as atheroembolism, renal artery or aortic dissection, renal artery rupture, and contrast-induced nephropathy.^8^ Early diagnosis and treatment reduce the ischemic injury.^3^ Controversy exists regarding the benefit of using these therapeutic options after prolonged ischemia. One study reported ischemia for >2 hours results in a 30% to 50% decrease in the long-term recovery from baseline.^9^ In a study by Silverberg et al,^3^ 13 of 42 patients with acute RAO were treated with catheter-directed thrombolysis. Of these 13 treated patients, 4 suffered ischemia of 72 to 96 hours, with successful revascularization and mild improvement of creatinine clearance between discharge and last follow-up.^3^ To the best of our knowledge, we report the second case of a patient with acute renal failure secondary to RAO with prolonged ischemia of 6 days who underwent successful thrombolysis with restoration of renal function. The other case was reported by Salam et al^2^ in a study of 10 cases of acute RAO treated by local infusion of fibrinolytic agents. The duration of ischemia in that case was also 6 days.^2^ One explanation for the recovery of renal function after prolonged ischemia is the presence of renal collateral circulation that can sustain renal viability via periureteric, peripelvic, and capsular branches.^10^ Although the aneurysmal sac had grown to its size 2 years before, it has been reasonably stable for the past 12 months. His current chronic kidney disease is prohibitive for selective arteriography, and his medical comorbidities make him a prohibitive candidate for an open procedure.

## Conclusions

Because of the significant morbidity of organ loss, RAO should be considered in patients who present with flank pain and anuria. The thrombolytic technique is a safe modality of therapy and, even for patients with prolonged ischemia, should be considered to salvage kidney function.

## Disclosures

J.C.Q.V. has participated in advisory board engagements and consulting with Mallinckrodt Pharmaceuticals, Bayer, and Travere Therapeutics and has been a member of a speaker bureau for Otsuka Pharmaceuticals. M.M.B.M., K.M.G.M., M.S.A.K., and W.C.S. have no conflicts of interest.
